# Association of polysialic acid serum levels with schizophrenia spectrum and bipolar disorder-related structural brain changes and hospitalization

**DOI:** 10.1038/s41598-023-29242-3

**Published:** 2023-02-06

**Authors:** Louisa Müller-Miny, Katharina Thiel, Susanne Meinert, Tim Hahn, Tilo Kircher, Igor Nenadić, Axel Krug, Felix Hufschmidt, Huan Liao, Harald Neumann, Udo Dannlowski, Jan D. Lünemann

**Affiliations:** 1 Department of Neurology With Institute of Translational Neurology, University Hospital Münster, 48149 Münster, Germany; 2 Institute for Translational Psychiatry, University of Münster, Münster, Germany; 3 Institute of Reconstructive Neurobiology, Medical Faculty and University Hospital of Bonn, University of Bonn, Bonn, Germany; 4 Department of Psychiatry and Psychotherapy, University Marburg, Marburg, Germany; 5 Marburg Center for Mind, Brain and Behavior (MCMBB), Marburg, Germany; 6 Department of Psychiatry and Psychotherapy, University Hospital Bonn, Bonn, Germany; 7 Institute for Translational Neuroscience, University of Münster, Münster, Germany

**Keywords:** Biomarkers, Outcomes research, Biomarkers, Pathogenesis, Risk factors, Molecular neuroscience

## Abstract

Expression of polysialic acid (polySia) in the adult brain is enriched in areas of continuous neurogenesis and plasticity such as the hippocampus. Genome-wide association studies identified variants of glycosylation enzyme-encoding genes, required for the generation of polySia, to be associated with the development of schizophrenia and bipolar disorder. Here, we report that serum levels of polySia are increased in patients with schizophrenia spectrum disorder compared to patients with major depressive disorders or demographically matched healthy controls. Furthermore, elevated polySia serum levels are associated with structural hippocampal gray matter decline in schizophrenia spectrum and bipolar disorder. In patients with schizophrenia spectrum disorder, polySia serum levels correlate with the number, duration of disease-related hospitalizations, early retirement and medical leave as estimators of detrimental long-term disease trajectories. Our data show that polySia serum levels are linked to structural hippocampal brain changes in schizophrenia spectrum and bipolar disorders, and suggest a contribution of polySia to the pathophysiology of these diseases.

The glycocalyx of mammalian cells is decorated with terminal sialic acids or neuraminic acids, which comprise a family of monosaccharides with a nine-carbon backbone^1^. Unlike other sugars, sialic acid often forms linear homopolymer structures such as polysialic acid (polySia) with variable degrees of polymerization ranging from 10 up to 200 sugar residues. Sialyltransferases are enzymes that mediate addition of sialic acids to the underlying glycan. Most of these linkages are in an alpha configuration and are defined by which carbon of the acceptor glycan is connected to anomeric carbon of the transferred sialic acid (carbon 2), resulting in α2,3 or a α2,6 linkages. For polySia, sialic acids are added to a strain of other sialic acid molecules in α2,8 linkages^2^.

The central nervous system (CNS) has the highest concentration of sialic acids in the body^1,3^. Most sialic acids are incorporated into gangliosides, while α2,8-linked polySia are linked to glycoproteins such as neural cell adhesion molecule (NCAM), synaptic cell-adhesion molecule (SynCAM)^4^, neuropilin-2^5^ and E-selectin ligand 1^6,7^ expressed on neuronal and glial cells. While polySia plays an important role during brain development, postnatal expression is reduced in most brain regions except for areas of continuous neurogenesis and plasticity such as the hippocampus^8,9^. The motility and plasticity-facilitating functions of the negatively charged polySia support regulation of synaptogenesis, neurogenesis, cell proliferation and migration, cell adhesion and axon guidance^1^. Indeed, murine studies show promise of intraperitoneal polySia injection as a therapeutic treatment in hindering neurodegeneration^10^ and in transporting treatments across the blood-CNS barrier^11,12^.

Given the role of polySia in brain development, neurogenesis, inflammation and its association with hippocampal structures, it has been suggested that structural or functional impairments of polySia may contribute to the development of psychiatric disorders such as schizophrenia (SZ) and schizoaffective disorder (SZA) (hereafter summarized as schizophrenia spectrum disorder SZ/SZA), bipolar disorder (BD), or major depressive disorder (MDD)^13,14^. Indeed, genome-wide association studies (GWAS) identified variants of glycosylation enzyme-encoding genes of the peripheral blood to be associated with aforementioned disease conditions^15^. Genetic variants or loss-of-function mutations of the α2,8 polysialyltransferase, ST8 α-*N*-acetyl-neuraminide α-2,8-sialyltransferase 2 (ST8SIA2) were reported to be associated with the development of SZ^13,16,17^ and BD^17^. Expression of polySia-NCAM (polysialylated NCAM) appears to be reduced in patients with SZ^18–20^, and mice deficient in ST8SIA2 exhibit SZ-like behavioral abnormalities, including cognitive dysfunction, deficits in prepulse inhibition, and increased sensitivity to amphetamine-induced locomotion^21^.

Recently, increased serum levels of polySia-NCAM have been reported in patients diagnosed with SZ^22^. However, this study specifically investigated whether the correlation between polySia-NCAM serum levels and gray matter volume (GMV) is different in SZ patients compared with healthy controls (HC). In a highly selective prefrontal cortical area, namely in a cluster located in the brodmann area (BA) 46, a negative correlation was found between increased polySia-NCAM serum levels and GMV in SZ patients, whereas a positive correlation was found in HC. Thus, this study^22^ provided a first indication of a correlation between polySia-NCAM serum levels and structural brain changes. Additionally, structures such as the hippocampus, where polySia is physiologically found in adults, plays a role in neuronal plasticity and shows pathological abnormalities in schizophrenia, have not been studied^8,9,23^. Here, we determined serum levels of polySia in patients with SZ/SZA, BD, and MDD compared to healthy individuals (HC) and examined the relationship between the serum levels of polySia and structural brain changes and as well as clinical parameters of disease severity.

## Results

### PolySia serum levels are increased in patients with schizophrenia spectrum disorder

**Figure 1:**
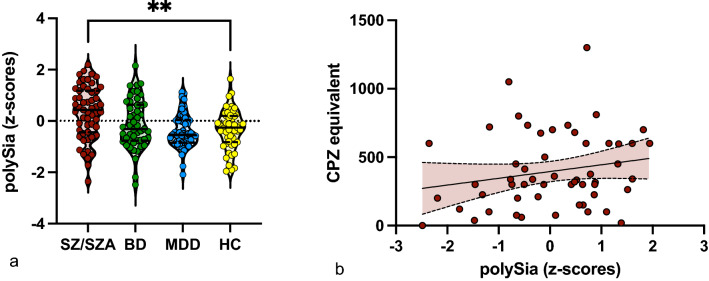
Serum levels of polySia are increased in SZ/SZA. Shown are mean serum levels of polySia in different patient subgroups (**a**) and comparison between the polySia serum level and the chlorpromazine equivalent (**b**). (**a**) Serum levels of polySia were increased in SZ/SZA compared to HC (*p < .05; **p < 0.01). (**b**) No correlation (*p* =  < .97) between the polySia serum levels and the chlorpromazine (CPZ) equivalent in the medication treated cohort of patients with SZ/SZA, BD and MDD. Error bars represent standard deviation from the mean.

We included 151 patients with the diagnosis SZ/SZA (*n* = 55), BD (*n* = 49), MDD (*n* = 49), and 51 healthy controls (HC), demographically matched for age and gender (Table 1). Identical protocols were used for sampling and processing biomaterial including serum for patients and HC and for obtaining clinical and magnetic resonance imaging (MRI) data in patients. Serum levels of soluble polySia were increased in patient group with SZ/SZA (*p* = 0.002) compared to HC. Interestingly, polySia serum levels were unchanged in patients with BD and MDD compared to HC (Fig. 1a). SZ/SZA patients also showed higher polySia serum levels compared to MDD patients (*p* = 0.003), while there was no significant difference between SZ/SZA and BD patients (*p* = 0.115).

**Table 1:** Demographic and clinical characteristics of the study sample.

	HC (n = 51)	MDD (n = 49)	BD (n = 49)	SZ/ SZA (n = 55)
Sociodemographic characteristics				
Age, M ± SD	37.29 ± 12.36	35.80 ± 11.41	41.88 ± 11.83	36.77 ± 11.78
Sex (male/female)	25/26	23/26	23/26	26/29
Years of education, M ± SD	13.55 ± 2.7	13.14 ± 2.39	14.65 ± 2.79	12.75 ± 2.75
Clinical characteristics and questionnaires				
Number of lifetime depressive episodes, M ± SD	N/A	5.04 ± 10.64	5.58 ± 4.64	3.84 ± 3.56
Lifetime duration of depressive episodes (months), M ± SD	N/A	58.17 ± 109.9	38.87 ± 70.74	65.78 ± 99.19
Number of lifetime manic episodes, M ± SD	N/A	N/A	5.00 ± 9.46	2.64 ± 3.27
Lifetime duration of manic episodes (months), M ± SD	N/A	N/A	12.46 ± 25.48	7.89 ± 9.70
Number of lifetime hospitalizations, M ± SD	N/A	1.32 ± 2.28	2.96 ± 3.02	4.20 ± 2.94
Lifetime duration of hospitalization (weeks), M ± SD	N/A	9.13 ± 17.77	23.60 ± 23.53	41.97 ± 52.68
Lifetime duration of medical leave (months), M ± SD	N/A	5.82 ± 8.52	12.94 ± 15.01	11.53 ± 16.34
SAPS, M ± SD	0.14 ± 0.456	0.67 ± 2.17	1.43 ± 2.16	49.08 ± 11.64
SANS, M ± SD	0.96 ± 2.55	7.86 ± 8.8	5.65 ± 7.2	14.11 ± 11.27
CPZE	N/A	12.65 ± 49.87	99.90 ± 218.1	343.8 ± 295.9
Smoker (%)	0	0	0	20
BMI (M ± SD)	25.33 ± 4.11	26.01 ± 5.80	27.04 ± 5.84	27.72 ± 5.23

*BD* bipolar disorder; *BMI* body mass index (Italic font), *CPZE* chlorpromazine equivalent; *HC* healthy controls; *MDD* major depressive disorder; *SANS* scale for the assessment of negative symptoms; *SAPS* scale for the assessment of positive symptoms; *SD* standard deviation; *SZ* schizophrenia; *SZA* schizoaffective disorder; *n* number; *n/a* not applicable.

A total of 66/153 (43.13%) of patients with SZ/SZA, BD and MDD received antipsychotic medication. 51 of the BD (*n* = *17*), SZ/SZA (*n* = *30*) and MDD (*n* = *4*) patients were treated with atypical antipsychotics while 3 received typical antipsychotics in the SZ/SZA cohort, 12 patients (SZ/SZA: *n* = *1*; BD: *n* = *11*) were treated with lithium. Correlations in the medication-treated cohort with the chlorpromazine equivalent (CPZE), as an equivalent for the cumulative potency of the antipsychotic drugs, did not detect any significant correlations between polySia serum levels and CPZE (Fig. 1b; *r* = 0.206; *p* = 0.135), indicating that antipsychotic medication unlikely contributes to increased serum levels of polySia in patients. Additionally, eight patients received tricyclic antidepressants (TCA), one received an norepinephrine reuptake inhibitor (NaRI), three received norepinephrine–dopamine reuptake inhibitor (NDRI), 29 were treated with selective serotonin reuptake inhibitors (SSRIs), 22 received serotonin–noradrenaline reuptake inhibitors (SNRIs) and seven received noradrenergic and specific serotonergic antidepressants (NaSSAs). Correlations with the Medindex composite score showed no significant correlation within the medication treated cohorts (Fig. S1; r = 0.037; *p* = 0.689) suggesting no additional contribution of other psychiatric medication to the increased polySia serum levels.

Thus, compared to healthy individuals, polySia serum levels were increased in patients with SZ/SZA, but not in BD and MDD. PolySia serum levels were not related to antipsychotic or other psychiatric medication.

### Elevated polySia levels correlate with hippocampal gray matter decline

To determine whether patient groups differ from HC in hippocampal and prefrontal GMV and whether polySia levels are associated with these potential brain structural changes, we performed voxel-based morphometry (VBM). Due to insufficient quality of images, *n* = 6 subjects (*n*_MDD_ = 2, *n*_BD_ = 1, *n*_SZ/SZA_ = 3) had to be excluded for these analyses. The region of interest (ROI) analyses of the bilateral hippocampal region revealed a significantly decreased GMV in SZ/SZA patients as well as in BD patients compared with HC, whereas MDD patients did not differ from HC (*p*_*tfce-FWE*_ = 0.088). Comparing SZ/SZA patients with HC, there was a significant reduction in GMV in SZ/SZA in four clusters in the bilateral parahippocampal region, mainly in the left hemisphere (left: x = − 18, y = − 12, z = − 27, *k* = 2146, *p*_*tfce-FWE*_ = 0.001; x = -30, y = − 20, z = − 27, *k* = 1, *p*_*tfce-FWE*_ = 0.046; right: x = 20, y = − 24, z = − 20, *k* = 858, *p*_*tfce-FWE*_ = 0.012; x = 20, y = − 33, z = − 8, *k* = 2, *p*_*tfce-FWE*_ = 0.042). BD patients showed decreased GMV in comparison to HC in two clusters in the bilateral parahippocampal region (left: x = -21, y = -26, z = -16, *k* = 1112, *p*_*tfce-FWE*_ = 0.002; right: x = 21, y = − 24, z = − 18, *k* = 1025, *p*_*tfce-FWE*_ = 0.006).

The ROI analysis of the BA 46 region revealed a significant reduction in GMV in SZ/SZA patients compared with HC, whereas MDD and BD patients did not differ from HC (MDD: *p*_*tfce-FWE*_ = 0.258; BD: *p*_*tfce-FWE*_ = 0.765). SZ/SZA patients showed significantly reduced GMV in four clusters in the bilateral BA 46 (left: x = -52, y = 30, z = -21, *k* = 75, *p*_*tfce-FWE*_ = 0.018; x = − 45, y = 20, z = 26, *k* = 5, *p*_*tfce-FWE*_ = 0.049; right: x = 51, y = 36, z = 20, *k* = 271, *p*_*tfce-FWE*_ < 0.001; x = 51, y = 22, z = 28, *k* = 31, *p*_*tfce-FEW*_ = 0.002).

The exploratory analysis of the correlation between hippocampal GMV and polySia serum concentrations revealed a significant negative association between these two measures in patients with BD and SZ/SZA in three clusters in the bilateral parahippocampal region (left: x = − 15, y = − 36, z = − 15, *k* = 80, *p*_*uncorrected*_ = 0.006, *r* = − 0.250; x = − 33, y = -27, z = − 6, *k* = 271, *p*_*uncorrected*_ = 0.015, *r* = − 0.195; right: x = 14, y = − 40, z = − 6, *k* = 189, *p*_*uncorrected*_ = 0.009, *r* = − 0.237).

**Figure 2:**
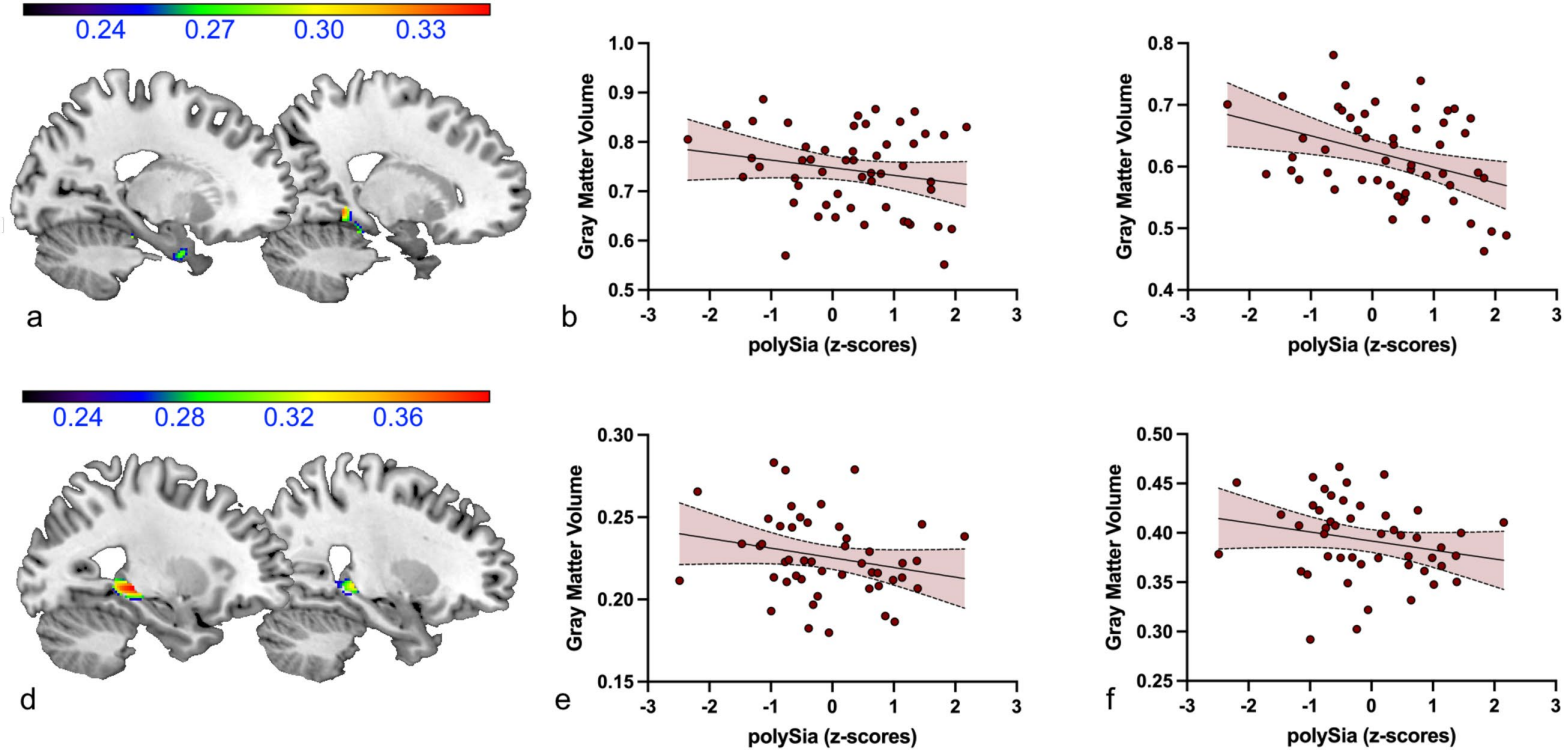
Negative association between polySia serum levels and bilateral parahippocampal gray matter volume in patients with SZ/SZA (**a–c**) and BD (**d–f**) (**a**) Sagittal views (x =  − 20, x = 18) of the significant clusters depicting gray matter volumes correlating with polySia levels in SZ/SZA patients. (**b**) Scatter plot depicting the negative correlation (*r* =  − .199, *p* = .025) of polySia levels and the peak voxel value (x =  − 20, y = 0, z =  − 32) of the cluster shown in A on the left. (**c**) Scatter plot depicting the negative correlation (*r* =  − .372, *p* = .010) of polySia levels and the peak voxel value (x = 18, y = -42, z = −6) of the cluster shown in A on the right. (**d**) Sagittal views (x = -21, x = 26) of the significant clusters depicting gray matter volumes correlating with polySia levels in BD patients. (**e**) Scatter plot depicting the negative correlation (*r* =  − .231, *p* = .004) of polySia levels and the peak voxel value (x =  − 27, y =  − 30, z =  − 3) of the cluster shown n in (**d**) on ther left. (**f**) Scatter plot depicting the negative correlation (*r* =  − .220, *p* = .013) of polySia levels and the peak voxel value (x = 26, y =  − 32, z =  − 2) of the cluster r shown in (**d**) other right. Color bars: negative correlation coefficient − *r*.

When only SZ/SZA patients were considered, two significant clusters in bilateral parahippocampal gray matter emerged (left: x = − 20, y = 0, z = − 32, *k* = 67, *p*_uncorrected_ = 0.025, *r* = − 0.199; right: x = 18, y = − 42, z = − 6, *k* = 69, *p*_*uncorrected*_ = 0.010, *r* = − 0.372, see Fig. 2a–c).

In BD patients, the regression analysis also revealed a significant negative association with polySia levels in the bilateral parahippocampal region in two clusters (left: x = − 27, y = − 30, z = − 3, *k* = 446, *p*_*uncorrected*_ = 0.004, *r* = − 0.231; right: x = 26, y = − 32, z = − 2, *k* = 187, *p*_*uncorrected*_ = 0.013, *r* = − 0.220, see Fig. 2d–f).

In patients with SZ/SZA, the exploratory analysis of the correlation between prefrontal GMV (BA 46) and polySia levels revealed no significant clusters using a threshold of *p*_*uncorrected*_ < 0.05 and *k* = 50.

Thus, the GMV of the bilateral parahippocampal region was reduced in patients with SZ/SZA and BD compared to HC. In addition, a negative correlation between the polySia serum levels and parahippocampal GMV was observed in these patients. Patients with SZ/SZA also showed a significant GMV reduction in the prefrontal region BA 46, but there was no significant association between GMV and polySia levels.

### Number and duration of hospitalizations correlate with poySia serum levels in SZ/SZA

We next investigated whether increased polySia serum levels are associated with clinical parameters of disease phenotype and severity in patients with SZ/SZA and BD. In patients with SZ/SZA, polySia serum levels did not correlate with clinical scores for acute positive (scale for the assessment of positive symptoms, SAPS: *r* = 0.107; *p* = 0.258)^24^ or negative symptoms (scale for the assessment of negative symptoms; SANS: *r* = − 0.109; *p* = 0.254)^25^. To determine effects relevant for long-term disease severity, frequency and duration of disease-related hospitalizations were assessed. Additionally we assessed medical leave and disease related retirement.

**Figure 3:**
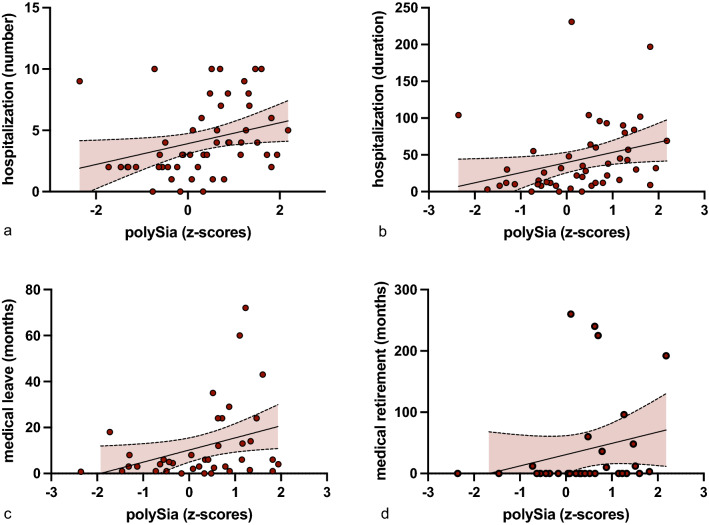
PolySia serum levels correlate with longterm disease trajectories. Correlation of polySia serum levels in SZ/SZA patients with (**a**) the number of hospitalization (*p* =  < .001)) (**b**) and duration of hospitalizations in months (*p* < .001),  (**c**) medical leave (*p* = .012) and  (**d**) early retirement due to medical reasons (*p* = .014).

In patients with SZ/SZA, polySia serum levels correlated with the number of hospitalizations (*r* = 0.447; *p* < 0.001) and the length of hospitalization (*r* = 0.467; *p* < 0.001)(Fig. 3). Additionally in patients with SZ/SZA medical leave (*r* = 0.362; *p* = 0.012) and the disease related retirement (r = 0.396; *p* = 0.014) (Fig. 3) were correlated with polySia serum levels. In patients with BD and MDD there was no correlation with clinical parameters such as length and number of hospitalizations.

Thus, increased polySia serum levels in SZ/SZA patients correlated with longterm dieases trajectories such as the number, length of hospitalization, medical leave and dieases related retirement.

## Discussion

In this study, we determined serum levels of soluble polySia in patients with a broad spectrum of psychotic and affective neuropsychiatric diseases as well as demographically matched HC. PolySia serum levels were specifically elevated in patients with SZ/SZA. Furthermore, elevated polySia levels were associated with hippocampal gray matter decline in SZ/SZA as well as BD, and also correlated in SZ/SZA with the number and duration of related hospitalizations, medical leave and disease related retirement as estimators of detrimental long-term disease trajectories.These data suggest that polySia might contribute to the pathophysiology of SZ/SZA and to, a lesser extend, of BD.

Soluble brain-derived molecules such as neurofilament light chain (NfL) protein can be quantified in serum and correlate with pathological processes confined to the central nervous system^26–28^. While polySia can be expressed broadly on various cell types, neuronal cell membranes are specifically enriched with polySia^29^ and our findings corroborate a previous investigation on elevated serum levels of polySia-NCAM in SZ patients^22^ providing further evidence for correlation between the pathophysiology of SZ/SZA and soluble polySia serum levels.

In contrast to the aforementioned study, we determined polySia levels independent of its protein carrier. This difference in the detected glycoconjugates may also underlie some of the observed discrepancies between these studies^22^, such as correlation with brain structural changes and clinical symptoms. Some of the measured polySia attached to proteins other than NCAM might be released by e.g. myeloid cells such as microglia, macrophages or other immune cells^30^, that are dysregulated in schizophrenia^30–32^.

In contrast to higher levels of polySia-NCAM^22^ and soluble polySia in SZ, numbers of polySia-positive cells and polySia immunoreactivity was reported to be decreased in hippocampal and prefrontal cortex regions of postmortem brains derived from SZ patients^18,19^. In microglia, shedding of sialylated proteins^33^ occurs upon activation^7,34^, and desialated microglia could facilitate further immune cell and complement activation^35^. Indeed, the desialyation of microglia due to inflammatory activation^6,7^ and the sensing of the desialylation by the alternative complement pathway^36^ could further support the role of neuroinflammation in schizophrenia^37^ especially as neuron-microglia interactions and changes in complement have been implicated in the pathogenesis of schizophrenia^38,39^. Similarly, shedding of polySia-NCAM and other polysialylated structures from neuronal cell surfaces^40^ that relate to neural activation or dysfunction, could subsequently lead to increased soluble serum levels of polySia. Hyposialylated neurons could be attacked and removed by complement and/or microglia, leading to reduced neuronal cell numbers^41^. This hypothesis is supported by the inverse correlation of soluble polySia serum levels with (para-)hippocampal GMV in patients with SZ/SZA.

Identification of validated serum biomarkers predicting disease course, severity and treatment benefits in patients with schizophrenia spectrum disorders could foster the development of precision medicine. Despite encouraging preliminary data on peripheral biomarkers, most candidates could not be confirmed and validated in subsequent studies. Inconsistent results might partly be explained by the clinical heterogeneity of disease conditions comprising different traits and disease states. Our finding that polySia serum levels are increased independent on a positive or negative SZ disease state, but are indicative for hospitalization rates suggests that soluble polySia levels should be further explored for their potential merit to reflect or even predict disease severity and quality of life measures in patients with SZ/SZA.

The observation that polySia serum levels are elevated in SZ/SZA and correlate with hippocampal GMV in SZ/SZA and BD, but not in MDD is interesting but also biologically plausible for a couple of reasons. First, MDD is a more prevalent and clinically as well as biologically a much more heterogeneous disorder with significantly lower heritability rates (up to 70–80% in SZ/SZA and BD compared to 30% in MDD). Second, in MDD, much larger influences of environmental risk factors such as childhood maltreatment experiences have been reported^42,43^. Third, in GWAS studies, genetic variation relevant to polySia has been found in SZ/SZA and BD, but not in MDD^2,16,44^. Fourth, while there is evidence that brain structural correlates of MDD share similarities with those found in SZ/SZA and BD^45^, and also hippocampal volume loss has been reported in previous studies on MDD in the past^46,47^, the effect sizes reported for MRI-derived structural brain alterations in MDD are generally lower compared to effect sizes reported in SZ/SZA and BD^48^. This might also explain the lack of a significant hippocampal volume reduction in the present MDD patient cohort. Furthermore, it has been reported that hippocampal volume loss in major depression might be rather a function of detrimental environmental experiences (childhood maltreatment) than the diagnosis itself^45^.

Limitations of our study include the retrospective cross-sectional design as well as the sample size. Prospective longitudinal studies including measurements of polySia in cerebrospinal fluid will be necessary to further validate our findings and to further establish a causal relationship between polySia and SZ/SZA and BD. While evidence suggests cross-talk of polySia^30,36,49^, possibly via an active transport mechanism^11,12^, over the blood-CNS barrier, further studies are necessary to better quantify this cross-talk. We have not seen any significant effect of antipsychotic or antidepressant medication on the polySia serum level, but we are aware that this could be a potential confounder. There is almost no literature on the effect of antipsychotic drugs on polySia expression, except for three murine studies showing a local increase of polySia-NCAM in the prefrontal cortex of mice and rats (1) when treated with chlorpromazine, (2) repeated risperidone^50,51^ or (3) olanzapine^52^ which could not be replicated in other regions indicating a region specific remodeling but not as a general effect of antipsychotic treatment. Additionally, when correlating the chlorpromazine equivalents, which is commonly used to quantify the antipsychotic potency, there was no significant correlation in our study. However, further studies with treatment naive patients would be valuable in a prospective study design.

Our data suggests, that soluble serum polySia might be a marker of the largely discussed trait like pattern^53,54^ in patients with schizophrenia spectrum disorder as it shows correlations with long term disease trajectories and changes in brain structure rather than short term state markers such as psychosis. Further prospective studies with treatment naïve and high-risk patients for a severe disease progression would be required to further investigate the state vs. trait hypothesis and to validate polySia as a biomarker in SZ/SZA patients.

In summary, our study identified increased serum levels of polySia to be associated with the diagnosis of schizophrenia spectrum disorder, with clinical markers of its detrimental disease course, as well as brain structural correlates of schizophrenia spectrum and bipolar disorder. This data strengthen the concept that either polySia synthesis, function or shedding contribute to the pathophysiology of SZ/SZA and suggest that the neuronal sialylation-desialylation machinery should be explored for its potential therapeutic merit in psychotic disorders. The results provide incentive to conduct larger, prospective studies to further elucidate the validity of soluble polySia levels in the serum as clinically useful biomarker in neuropsychiatric diseases.

## Methods

### Study participants

Serum concentrations of polysialic acid (polySia) were measured cross-sectionally in a cohort of patients with SZ and SZA (*n* = 55), BD (*n* = 49), MDD, (*n* = 49) and HC (*n* = 51). Subjects were matched for age, sex, and years of education. Sociodemographic and clinical characteristics of the sample groups are shown in Table 1. Data and samples comprised in the Marburg-Münster Affective Disorders Cohort Study (MACS; see^55^ for the general description of the study). Data was collected at two scanning sites—the University of Marburg and the University of Münster (see reference^56^ for further details on the magnetic resonance imaging (MRI) quality assurance protocol). Participants were recruited via newspaper advertisements or in psychiatric hospitals. The study was approved by the Ethics Committees of the Medical Faculties, University of Marburg (AZ: 07/14) and University of Münster (2014-422-b-S), in accordance with the Declaration of Helsinki. All subjects provided written informed consent prior to participation and received financial compensation. All participants completed the Structured Clinical Interview for DSM-IV^57^ to verify a lifetime psychiatric diagnoses or the lack thereof. For the HC group, any lifetime psychiatric disorder according to the SCID-I as well as any intake of psychotropic medication resulted in exclusion from the study. General exclusion criteria were no imaging data, common contraindications against MRI, a non caucasian background, medicaton with benzodiazapene or other sleep-inducing drugs, a lifetime diagnosis of substance dependence, low compliance, traumatic head injury, neurological, inflammatory, infectious- and immunoassociated diseases as well was malign neoplasia to exclude other diseases causing an increase in polySia.

In the medication-treated cohort we quantified the antipsychotic drugs by quantifiying the chlorpromazine equivalent (CPZE) of each antipsychotic drug. The total daily dose of a medication expressed in milligrams of Chlorpromazine per day was defined as a CPZE^58^.

Addititonally to further analyse associations with other psychiatric medications we calculated the Medication Load Index (MedIndex), as a composite measure of individual total medication load using a strategy described previously^59,60^ In brief: A value between 0 and 2 was assigned to each psychopharmacological agent depending on whether that medication was absent (= 0), the dose was equal to or lower than the average dose (low = 1), or the dose was higher than average dose (high = 2). The average dose was defined through the daily dose intake recommended by the Physician’s-Desk Reference^61^. The sum of these values was calculated resulting in the MedIndex and indicates the number and daily dose of all psychopharmacological medications currently taken by the participant.

### Quantification of serum polysialic acid levels

A 96 well ELISA-plate (Nunc MaxiSorp, ThermoFisher 44-2404-21) was coated with a capture antibody (Rat-anti polySia/CD56 IgM clone 12F8, 1:500, stock conc.: 0.5 mg/ml, 556,325, BD Pharmingen, New York, USA, in a coating buffer), incubated overnight, washed with a washing buffer and blocked for with a 0,5% fish gelantine containing blocking buffer. The standard curve was established with α2,8-linked polySia (~ 25–50 kDa with average molecular weight of 40 kDa). The polysialic acid (α2,8-linked N-acetylneuraminc acid ~ 25–50 kDa) was obtained from cultures of Escherichia coli B2032/82 serotype K1 and purified as previously described^10^. Sera were obtained (as shown previously here:^55^and aliquots were stored at − 80 °C. For analysis, serum samples were thawed and diluted 1:10 in washing buffer. Then, 100 µl of the samples and the polySia standard were added to the wells and incubated overnight. All further steps consisted of the plate being washed with washing buffer (3 times) after each incubation period at room temperature. A detection antibody (mouse anti polySia/CD56 IgG2a clone 735, Novus Biologicals, Littleton, Colorado, USA, NBP252710; 1:200, stock conc. 0.2 mg/ml) was added in blocking buffer and incubated for 1 h. The biotinylated antibody (Biotin-SP-conjugated rat-anti-mouse IgG AffiniPure, Dianova, Hamburg, Germany, 415-065-166, 1:10,000, stock conc. 2 mg/ml) was added with a further incubation time of 1 h. The Streptavidin-HRP (Upstate, Sigma-Aldrich, Taufkirchen, Germany, 18-152) conjugate was added in a concentration of 1 µg/ml and incubated for 1 h. Detection agent tetramethylbenzidine (Sigma-Aldrich, T0440) was added and incubated for 10 min after which the reaction was stopped with 1 M HCl. The samples were than measured in a Tecan Infinite 200pro at a wavelength of 450 nm with a reference at 620 nm. The samples were run in duplicates with a standard and interassay control on every plate. The aliquots used have not been repeatedly frozen/thawed prior to this study. The intraassay variance was less than 9.8% and the interassay variance was 28.3%. Data was normalized through a z-score, by subtracting the mean value of all samples from one assay from the individual mean sample value and dividing this by the standard deviation.

### MRI data acquisition

T1-weighted high-resolution anatomical data were acquired with 3 T MRI scanners (Marburg: 12-channel head matrix Rx-coil; Tim Trio, Siemens, Erlangen, Germany; Münster: 20-channel head matrix Rx-coil; Prisma, Siemens, Erlangen, Germany). A three dimensional fast gradient echo sequence (MPRAGE) was used with the following parameters: field of view (FOV) = 256 mm, 176 sagittal slices, TR = 1900 ms, TE = 2.26 ms, inversion time = 900 ms, FA = 9°, voxel size = 1 × 1 × 1mm^3^ (Marburg) and FOV = 256 mm, 192 sagittal slices, slice gap 0 mm, TR = 2130 ms, TE = 2.28 ms, inversion time = 900 ms, FA = 8°, voxel size = 1 × 1x1mm^3^ (Münster). The body coil at the scanner site in Marburg was exchanged during data acquisition, resulting in three different scanner settings for all analyses (Marburg body- coil pre, Marburg body- coil post and Münster). Further details of the imaging procedures have been extensively described elsewhere^56^.

### Voxel- based morphometry (VBM)

T1-weighted images were preprocessed using the VBM8-toolbox (http://dbm.neuro.uni-jena.de/vbm, Version r445) with a default processing pipeline. Briefly, images were bias-corrected, tissue-classified, and normalized to MNI-space using linear and non-linear transformation (including high-dimensional DARTEL-normalization). As the non-linear modulation directly corrects for different head sizes, total intracranial volume is not necessary as covariate in second level statistical models. Modulated gray matter segments were smoothed with an 8 mm full width half maximum Gaussian kernel. All images passed visual quality control, checking for artifacts and image quality by experienced researchers. Quality checks were performed following the quality assurance protocol by the MACS-study^56^.

### Statistical analysis

Data was analysed using GraphPad Prism 9. To examine differences in the polySia levels between the groups, an ANOVA with post hoc Dunnetts multiple comparisons test was calculated. To investigate associations between polySia and clinical parameters in SZ/SZA and BD the one-sided Spearman's rank correlation coefficient was determined. A *p* < 0.05 was considered significant. Statistical analyses of the MRI data were performed using Statistical Parametric Mapping (SPM12, v7771, Wellcome Department of Cognitive Neurology, London, UK) using full factorial models, with age, sex and scanner settings (Münster, Marburg body-coil pre/post) included as nuisance variables in all models. First, to investigate reductions in hippocampal and prefrontal GMV in each patient group compared with HC, pairwise t-contrasts (HC > MDD, HC > BD, HC > SZ/SZA) were performed in region of interest (ROI) analyses of the bilateral hippocampal region and BA 46, respectively. The mask for the bilateral hippocampal region was defined according to the AAL-atlas^62^ which is integrated in the WFU-pickatlas^63^, including the parahippocampus and hippocampus. The mask for the bilateral BA 46 was also defined using the WFU-pickatlas.

We applied threshold- free cluster enhancement (TFCE) implemented in the TFCE-toolbox (http://dbm.neuro.uni-jena.de/tfce, Version 232) with significance threshold using a conservative family-wise error (FWE) correction of *p* < 0.05 (with 5000 permutations per test).

Second, to examine associations of hippocampal or prefrontal GMV and polySia levels in all patients that showed GMV differences from HC, polySia concentrations were included in the model as the covariate of interest. According the existing findings, we analysed negative correlations of polySia levels with GMV in ROI analyses of the bilateral hippocampal region and BA 46. Representing exploratory analyses, an uncorrected voxel-level threshold of *p* < 0.05 and a cluster threshold of *k* = 50 voxel was applied.

## Supplementary Information

Supplementary Information.

## Data Availability

The dataset generated during or analysed during the current study are available from the corresponding author on reasonable request.
