# Supplementary material for: Association of polysialic acid serum levels with schizophrenia spectrum and bipolar disorder-related structural brain changes and hospitalization
Source: Sci Rep. 2023 Feb 6;13:2085. doi: 10.1038/s41598-023-29242-3 (PMC9902615; doi:10.1038/s41598-023-29242-3)
Supplement: Supplementary file 1 — Supplementary Information. [file 41598_2023_29242_MOESM1_ESM.pdf]

Supplementary Figure 1

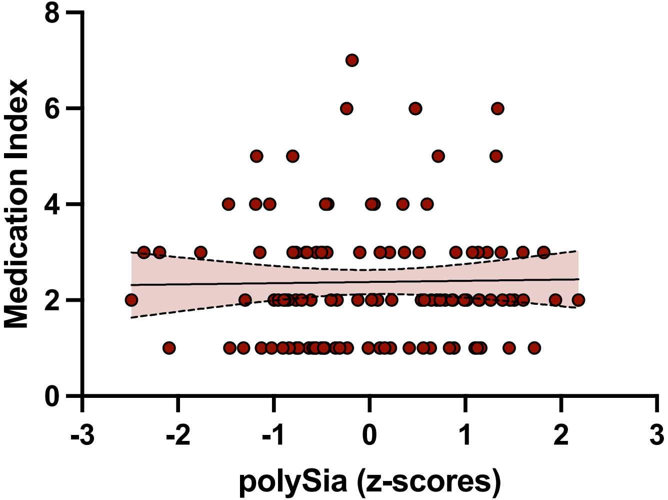

Supplementary Figure 1: correlation of the Medication Load Index (MedIndex) in the Medication treated cohort with polySia serum levels in all patients *(p=.803)*
